# Supplementary material for: The Temperature-Associated Effects of Rift Valley Fever Virus Infections in Mosquitoes and Climate-Driven Epidemics: A Review
Source: Viruses. 2025 Feb 1;17(2):217. doi: 10.3390/v17020217 (PMC11860320; doi:10.3390/v17020217)
Supplement: Supplementary file 1 [file viruses-17-00217-s001.zip › viruses-3370592-supplementary-xml.pdf]

viruses MDPI

Supplementary table S1: Important mosquito species and commonly transmitted viruses within the order *Bunyavir*ales

Arbovirus	Geographic range	Common vector spp.	Associated drivers of TOT/vector capacity	Disease	Genetic reassortment	Ref.
LACV	North America	*Ae. aegypti*<br>*Ae. albopictus*<br>*Ae. triseriatus*<br>*Ae. japonicus*	variations in vector genetics<br>venereal transmission, transmission barriers; vector competence, quantitative trait loci, M segment critical for TOT, amino acid residues in NSm, salivary gland infection during larval and pupal development critically important<br>possibly involved in virus maintenance and transmission	Human: encephalitis<br>infect small mammals	LACV and JCV co-infect *Ae. albopictus* mosquitoes with all potential reassortant genotypes transmitted to suckling mice, where the reassortant viruses contain both the highly neuroinvasive JCV genetic background and the LACV M segment	[1-12]
JCV	North America	*Ae. triseriatus*<br>*Ochlerotatus (Oc.) stimulans*<br>*Ae. squamiger*	established in germline tissue pre-eclosion<br>possible venereal transmission<br>water temperature of *Ae. squamiger* larvae	Human: neuroinvasive in humans causing encephalitis, fever	segment reassortment, or genetic shift, between two distinct LACV genotypes has been shown to occur in mosquitoes	[13-16]
TAHV	Asia, Europe	*Ae. vexans*<br>*Ae. caspius*<br>*Ae. aegypti*	climate pattern via diapausing eggs<br>variations in virus/vector genetics<br>transovarial transfer of the virus by germinal cells	Human: neuroinvasive, mostly flu-like, fever	reassortants between poorly pathogenic TAHV strain and a virulent LACV strain in BHK-21 cells (further ascertained for neuroinvasion in suckling mice), with neuroinvasiveness predominantly localized to the M segment	[17-20]

SSHV	North America, Asia	*Ae. communis*<br><br>*Ae. triseriatus*	transovarial transmission likely important in wild-caught *Aedes* immatures<br><br>vertical and venereal routes	Human: encephalitis, fever<br><br>Infect animals in the wild	*Ae. triseriatus* mosquitoes shown to more efficient at dissemination and transmitting LACV-SSHV reassortant viruses with the LACV M segment	[7, 21-28]
INKV	Asia, Europe	*Oc. communis*<br><br>*Ae. communis, Ae. hexodontus, Ae. punctor, Ae. cinereus, Cx. torrentium, Cx. pipiens*	detected in mosquito larvae indicating that these viruses could be maintained via vertical transmission	Human: neuroinvasive, encephalitis, affect mammals	It is suggested that a potential reassortment event in the INKV L segment occurred resulting from JCV	[29-32]
CEV	North America	*Ae. dorsalis*<br><br>*Ae. melanimon*	gonotrophic cycle, survival and development time, transmission barriers; vector competence, larval rearing temperatures	Human: encephalitis, fever		[33-35]
GROV	South America	*Culex* and *Anopheles spp.*		Humans and animals	hypothesized to be a reassortant virus members of the serogroup, however no conclusive evidence has been determined	[36-40]
CVV	North America	primary vectors remain unknown despite several mosquito species implicated in transmission	vector-vector transovarial transmission has been demonstrated only in *Culiseta inornata*	Sheep, cattle: congenital defects	between lineages I and II, reassortants have been verified; possible impact on vector competence	[41-45]
AKAV	Asia, Africa, Australia & Oceania	mosquitoes and biting midges implicated as putative vectors	inability to demonstrate TOT from a single in *Culicoides brevitarsis*	Reproductive failure and fetal deformities in ruminants Cattle: abortion and congenital defects	genetic reassortment event among field isolates is indicated in the S, M, and L RNA segments	[46, 47]
RVFV	Africa	*Ae. aegypti*<br><br>*Ae. circumluteolus*<br><br>*Ae. mcintoshi*<br><br>*Ae. lineatopennis*	NSm deletion, maturing oocytes via germinal cells<br><br>transmission barriers; vector competence<br><br>transmission barriers; vector competence, persistence through interepidemic periods, climate patterns/El Nino	Human: encephalitis, haemorrhagic fever, Domestic ruminants: necrotic hepatitis, haemorrhage, abortion	evidence of RVFV lineages have undergone intratypic reassortment during their evolutionary drive	[48-75]

		Ae. vexans	TOT detected in field samples: virus isolated from both male and female adult mosquitoes reared from larvae, pupae and eggs			
		Cx. pipiens	transmission barriers; vector competence			
		Cx. tarsalis	transmission barriers; accumulated antigen in the developing oocytes of the ovaries, subsequent blood feeding critical after initial exposure			
		Cx. poicilipes, Cx. quinquefasciatus, Cx. restuans, Cx. tritaenorhynchus	RVFV either isolated and or vector competence studies have demonstrated the vectorial capacity			
		Anopheles spp. including stephensi, arabiensis, cinereus, coustani, crucians				
		Mansonia spp. including africana, dyari, uniformis	associated with RVFV transmission			
		Eretmapodite spp. including quinquevittatus, subsimplicipes	evidence of vector competence for RVFV transmission			
TVTV	North America	*Ae. infirmatus*<br>*Ae trivittatus*	transovarial transmission has been demonstrated in TVT	neurological disease		[76, 77]
SAV	North America	*Ae. albopictus*	survival and development time, transmission barriers; vector competence, maternal inheritance, water temperature	Humans and animals		[78-80]
KEYV	North America	*Ae. atlanticus*	larval breeding habitats linked	mammals	past reassortment from unidentified progenitor strains to the reassorted genomes discovered in the M and S genome segments	[62, 77, 78, 81-85]
		Ae. albopictus	Vectorial capacity for TOT demonstrated			
		Ae. tormentor, Ae. infirmatus				
NRIV	Asia, Africa	*Ae. simpsoni*				[40, 86-88]

		An. gambiae, An. pharoensis, Cx. antennatus, Cx. poicilipes and Cx. tritaeniorhynchus	first isolated from male *Ae. simpsoni* mosquitoes reared from field-collected eggs.	Human: haemorrhagic fever		
BUNV	Africa, South America	Some *Aedes (Ae. aegypti* suggested as primary vectors) and *Culex spp.*		Human, livestock, birds	NRIV (a natural reassortant) reassorting from the M segment of BATV and the L and S segments of BUNV	[89, 90]
BATV	Asia, Europe, Africa	*Anopheles, Aedes Culex spp.*		Humans and animals		[30, 91, 92]
OROV	North and South America	relies primarily on its midge vector *(Culicoides paraensis)*, and vectors such as *Cx. quinquefasciatus, Ae. serratus*	No evidence of vertical transmission has been established	Human: febrile illness	reassortants of OROV include the Jatobal (JAT) and Iquitos (IQT) viruses, where JATV carries the S RNA and IQTV the S and L RNA of OROV	[93-96]
MBV	North America	*Ae. squamiger*	Transovarially transmitted	Human: febrile illness		[97]

La Crosse virus (LACV), Jamestown Canyon Virus (JCV), Tahyna virus (TAHV), Snowshoe hare virus (SSHV), Inkoo virus (INKV), California encephalitis virus (CEV), Guaroa virus (GROV), Cache Valley virus (CVV), Akabane virus (AKAV), Rift Valley virus (RVFV), Trivittatus virus (TVTV), San Angelo virus (SAV), Keystone virus (KEYV), Ngari virus (NRIV), Bunyamwera virus (BUNV), Batai virus (BATV), Oropouche virus (OROV).by

References

1. Gargan 2nd, T., et al., *Vector potential of selected North American mosquito species for Rift Valley fever virus.* The American journal of tropical medicine and hygiene, 1988. **38**(2): p. 440-446.
2. Beaty, B.J. and W.H. Thompson, *Delineation of La Crosse virus in developmental stages of transovarially infected Aedes triseriatus.* The American Journal of Tropical Medicine and Hygiene, 1976. **25**(3): p. 505-512.
3. Thompson, W.H., *Higher venereal infection and transmission rates with La Crosse virus in Aedes triseriatus engorged before mating.* The American Journal of Tropical Medicine and Hygiene, 1979. **28**(5): p. 890-896.
4. Reese, S.M., et al., *Identification of super-infected Aedes triseriatus mosquitoes collected as eggs from the field and partial characterization of the infecting La Crosse viruses.* Virology Journal, 2010. **7**: p. 1-27.
5. Graham, D., et al., *Selection of refractory and permissive strains of Aedes triseriatus (Diptera: Culicidae) for transovarial transmission of La Crosse virus.* Journal of medical entomology, 1999. **36**(6): p. 671-678.
6. Graham, D.H., et al., *Quantitative trait loci conditioning transovarial transmission of La Crosse virus in the eastern treehole mosquito, Ochlerotatus triseriatus.* Insect molecular biology, 2003. **12**(4): p. 307-318.
7. Schopen, S., M. Labuda, and B. Beaty, *Vertical and venereal transmission of California group viruses by Aedes triseriatus and Culiseta inornata mosquitoes.* Acta virologica, 1991. **35**(4): p. 373-382.
8. Miller, B.R., G. DeFoliart, and T. Yuill, *Vertical transmission of La Crosse virus (California encephalitis group): transovarial and filial infection rates in Aedes triseriatus (Diptera: Culicidae).* Journal of Medical Entomology, 1977. **14**(4): p. 437-440.
9. Hughes, M.T., et al., *Comparative potential of Aedes triseriatus, Aedes albopictus, and Aedes aegypti (Diptera: Culicidae) to transovarially transmit La Crosse virus.* Journal of medical entomology, 2006. **43**(4): p. 757-761.
10. Westby, K.M., et al., *La Crosse encephalitis virus infection in field-collected Aedes albopictus, Aedes japonicus, and Aedes triseriatus in Tennessee.* Journal of the American Mosquito Control Association, 2015. **31**(3): p. 233-241.
11. Tesh, R.B. and D.J. Gubler, *Laboratory studies of transovarial transmission of La Crosse and other arboviruses by Aedes albopictus and Culex fatigans.* The American journal of tropical medicine and hygiene, 1975. **24**(5): p. 876-880.
12. Cheng, L., et al., *Potential for evolution of California serogroup bunyaviruses by genome reassortment in Aedes albopictus.* The American journal of tropical medicine and hygiene, 1999. **60**(3): p. 430-438.
13. Berry, R., et al., *Evidence for transovarial transmission of Jamestown Canyon virus in Ohio.* 1977.
14. Boromisa, R.D. and P.R. Grimstad, *Virus-vector-host relationships of Aedes stimulans and Jamestown Canyon virus in a northern Indiana enzootic focus.* The American journal of tropical medicine and hygiene, 1986. **35**(6): p. 1285-1295.
15. Kramer, L.D., et al., *Vector competence of alpine, Central Valley, and coastal mosquitoes (Diptera: Culicidae) from California for Jamestown Canyon virus.* Journal of medical entomology, 1993. **30**(2): p. 398-406.
16. Beaty, B.J., et al., *Evolution of bunyaviruses by genome reassortment in dually infected mosquitoes (Aedes triseriatus).* Science, 1985. **230**(4725): p. 548-550.
17. Labuda, M., F. Ciampor, and O. Kozuch, *Experimental model of transovarial transmission of Tahyna virus in Aedes aegypti mosquitoes.* Acta virologica, 1983. **27**(3): p. 245-250.
18. Danielova, V. and J. Ryba, *Laboratory demonstration of transovarial transmission of Tahyna virus in Aedes vexans and the role of this mechanism in overwintering of this arbovirus.* 1979.
19. Moreau, J., P. Bihan-Faou, and G. Sinegre, *Tahyna virus transovarial transmission, trials in Aedes caspius.* 1976.
20. Janssen, R., et al., *Virulence of La Crosse virus is under polygenic control.* Journal of virology, 1986. **59**(1): p. 1-7.
21. McLean, D.M., et al., *Vector capability of Aedes aegypti mosquitoes for California encephalitis and dengue viruses at various temperatures.* Canadian Journal of Microbiology, 1974. **20**(2): p. 255-262.
22. Snyman, J., et al., *California Serogroup viruses in a changing Canadian Arctic: A review.* Viruses, 2023. **15**(6): p. 1242.

23. Beaty, B., et al., *Molecular basis of bunyavirus transmission by mosquitoes: role of the middle-sized RNA segment.* Science, 1981. **211**(4489): p. 1433-1435.
24. Beaty, B., et al., *Molecular basis of bunyavirus per os infection of mosquitoes: role of the middle-sized RNA segment.* Proceedings of the National Academy of Sciences, 1982. **79**(4): p. 1295-1297.
25. Borucki, M.K., et al., *Bunyavirus superinfection and segment reassortment in transovarially infected mosquitoes.* Journal of general virology, 1999. **80**(12): p. 3173-3179.
26. Jansen, S., et al., *Mosquitoes from Europe Are Able to Transmit Snowshoe Hare Virus.* Viruses, 2024. **16**(2): p. 222.
27. McLean, D., et al., *California encephalitis virus prevalence throughout the Yukon Territory, 1971-1974.* The American Journal of Tropical Medicine and Hygiene, 1975. **24**(4): p. 676-684.
28. Belloncik, S., et al., *Activity of California encephalitis group viruses in Entrelacs (province of Quebec, Canada).* Canadian journal of microbiology, 1982. **28**(6): p. 572-579.
29. Tingström, O., et al., *Detection of Sindbis and Inkoo virus RNA in genetically typed mosquito larvae sampled in Northern Sweden.* Vector-Borne and Zoonotic Diseases, 2016. **16**(7): p. 461-467.
30. Francy, D.B., et al., *Ecologic studies of mosquitoes and birds as hosts of Ockelbo virus in Sweden and isolation of Inkoo and Batai viruses from mosquitoes.* The American journal of tropical medicine and hygiene, 1989. **41**(3): p. 355-363.
31. Wesula Lwande, O., et al., *Inkoo virus: a common but unrecognized mosquito-borne virus in northern Europe.* Infectious Diseases Hub, 2017.
32. Hughes, H.R., et al., *Full genomic characterization of California serogroup viruses, genus Orthobunyavirus, family Peribunyaviridae including phylogenetic relationships.* Virology, 2017. **512**: p. 201-210.
33. Turell, M.J., W.C. Reeves, and J.L. Hardy, *Evaluation of the efficiency of transovarial transmission of California encephalitis viral strains in Aedes dorsalis and Aedes melanimon.* The American journal of tropical medicine and hygiene, 1982. **31**(2): p. 382-388.
34. Turell, M.J., W.C. Reeves, and J.L. Hardy, *Transovarial and trans-stadial transmission of California encephalitis virus in Aedes dorsalis and Aedes melanimon.* The American journal of tropical medicine and hygiene, 1982. **31**(5): p. 1021-1029.
35. Turell, M.J., J.L. Hardy, and W.C. Reeves, *Stabilized infection of California encephalitis virus in Aedes dorsalis, and its implications for viral maintenance in nature.* The American Journal of Tropical Medicine and Hygiene, 1982. **31**(6): p. 1252-1259.
36. Dunn, E.F., D.C. Pritlove, and R.M. Elliott, *The s rna genome segments of batai, cache valley, guaroa, kairi, lumbo, main drain and northway bunyaviruses: Sequence determination and analysis.* Journal of General Virology, 1994. **75**(3): p. 597-608.
37. Briese, T., A. Rambaut, and W.I. Lipkin, *Analysis of the medium (M) segment sequence of Guaroa virus and its comparison to other orthobunyaviruses.* Journal of general virology, 2004. **85**(10): p. 3071-3077.
38. Dégallier, N., et al., *As aves como hospedeiras de arbovírus na Amazônia Brasileira.* Boletim do Museu Paraense Emílio Goeldi. Nova série. Zoologia, 1992. **8**(1): p. 69-111.
39. Shope, R.E. and J.P. Woodall, *The epidemiology of diseases caused by viruses in Groups C and Guama (Bunyaviridae)*, in *Arboviruses*. 2019, CRC Press. p. 37-52.
40. Karabatsos, N., *American Society of Tropical Medicine and Hygiene, American Committee on Arthropod-borne Viruses, and and Rockefeller Foundation.* International Catalogue of Arboviruses, Including Certain Other Viruses of Vertebrates, 1985.
41. Dieme, C., et al., *Role of Anopheles mosquitoes in Cache Valley virus lineage displacement, New York, USA.* Emerging Infectious Diseases, 2022. **28**(2): p. 303.
42. Al-Heeti, O., et al., *Transfusion-transmitted Cache Valley virus infection in a kidney transplant recipient with meningoencephalitis.* Clinical Infectious Diseases, 2023. **76**(3): p. e1320-e1327.
43. Baker, M., et al., *Reassortant Cache Valley virus associated with acute febrile, nonneurologic illness, Missouri.* Clinical Infectious Diseases, 2021. **73**(9): p. 1700-1702.
44. Dunlop, J.I., et al., *Development of reverse genetics systems and investigation of host response antagonism and reassortment potential for Cache Valley and Kairi viruses, two emerging orthobunyaviruses of the Americas.* PLoS neglected tropical diseases, 2018. **12**(10): p. e0006884.

45. Corner, L.C., et al., *Cache Valley virus: experimental infection in Culiseta inornata.* Canadian journal of microbiology, 1980. **26**(3): p. 287-290.
46. Allingham, P. and H. Standfast, *An investigation of transovarial transmission of Akabane virus in Culicoides brevitarsis.* 1990.
47. Kobayashi, T., et al., *Genetic diversity and reassortments among Akabane virus field isolates.* Virus research, 2007. **130**(1-2): p. 162-171.
48. Kading, R.C., et al., *Deletion of the NSm virulence gene of Rift Valley fever virus inhibits virus replication in and dissemination from the midgut of Aedes aegypti mosquitoes.* PLoS neglected tropical diseases, 2014. **8**(2): p. e2670.
49. Bird, B.H., et al., *Complete genome analysis of 33 ecologically and biologically diverse Rift Valley fever virus strains reveals widespread virus movement and low genetic diversity due to recent common ancestry.* Journal of virology, 2007. **81**(6): p. 2805-2816.
50. Turell, M., K. Linthicum, and J. Beaman, *Transmission of Rift Valley fever virus by adult mosquitoes after ingestion of virus as larvae.* The American journal of tropical medicine and hygiene, 1990. **43**(6): p. 677-680.
51. Bergren, N.A., et al., *Laboratory demonstration of the vertical transmission of Rift Valley fever virus by Culex tarsalis mosquitoes.* PLoS Neglected Tropical Diseases, 2021. **15**(3): p. e0009273.
52. Linthicum, K., et al., *Rift Valley fever virus (family Bunyaviridae, genus Phlebovirus). Isolations from Diptera collected during an inter-epizootic period in Kenya.* Epidemiology & Infection, 1985. **95**(1): p. 197-209.
53. Iranpour, M., M. Turell, and L. Lindsay, *Potential for canadian mosquitoes to transmit rift valley fever virus1.* Journal of the American Mosquito Control Association, 2011. **27**(4): p. 363-369.
54. Sang, R., et al., *Rift Valley fever virus epidemic in Kenya, 2006/2007: the entomologic investigations.* The American journal of tropical medicine and hygiene, 2010. **83**(2 Suppl): p. 28.
55. Turell, M.J., et al., *Potential for North American Mosquitoes to Transmit Rift Valley Fever Virus1.* Journal of the American Mosquito Control Association, 2008. **24**(4): p. 502-507.
56. Jupp, P., et al., *The 2000 epidemic of Rift Valley fever in Saudi Arabia: mosquito vector studies.* Medical and veterinary entomology, 2002. **16**(3): p. 245-252.
57. McIntosh, B., * Jupp, PG,* Dos Santos, I.* and B. Barnard, *Vector studies on Rift Valley fever virus in South Africa.* South African Medical Journal, 1980. **58**(3): p. 127-132.
58. Seufi, A.M. and F.H. Galal, *Role of Culex and Anopheles mosquito species as potential vectors of rift valley fever virus in Sudan outbreak, 2007.* BMC infectious Diseases, 2010. **10**: p. 1-8.
59. Moutailler, S., et al., *Potential vectors of Rift Valley fever virus in the Mediterranean region.* Vector-borne and zoonotic Diseases, 2008. **8**(6): p. 749-754.
60. Asare, E.O., et al., *Mosquito breeding site water temperature observations and simulations towards improved vector-borne disease models for Africa.* Geospatial health, 2016. **11**(s1).
61. Turell, M.J. and W.S. Romoser, *Effect of the developmental stage at infection on the ability of adult Anopheles stephensi to transmit Rift Valley fever virus.* The American journal of tropical medicine and hygiene, 1994. **50**(4): p. 448-451.
62. Turell, M.J., et al., *Potential for mosquitoes (Diptera: Culicidae) from Florida to transmit Rift Valley fever virus.* Journal of Medical Entomology, 2013. **50**(5): p. 1111-1117.
63. Ratovonjato, J., et al., *Detection, isolation, and genetic characterization of Rift Valley fever virus from Anopheles (Anopheles) coustani, Anopheles (Anopheles) squamosus, and Culex (Culex) antennatus of the Haute Matsiatra region, Madagascar.* Vector-Borne and Zoonotic Diseases, 2011. **11**(6): p. 753-759.
64. Turell, M.J., et al., *Vector competence of selected African mosquito (Diptera: Culicidae) species for Rift Valley fever virus.* Journal of medical entomology, 2008. **45**(1): p. 102-108.
65. Logan, T., et al., *Isolation of Rift Valley fever virus from mosquitoes (Diptera: Culicidae) collected during an outbreak in domestic animals in Kenya.* Journal of medical entomology, 1991. **28**(2): p. 293-295.
66. Lutomiah, J., et al., *Blood meal analysis and virus detection in blood-fed mosquitoes collected during the 2006–2007 Rift Valley fever outbreak in Kenya.* Vector-Borne and Zoonotic Diseases, 2014. **14**(9): p. 656-664.

67. Clerc, Y. and P. Coulanges, *Rapport du laboratoire d'Arbovirus pour 1978.* Archives de l'Institut Pasteur de Madagascar, 1979. **47**: p. 64-68.
68. Rattanarithikul, R., et al., *Illustrated keys to the mosquitoes of Thailand III. Genera aedeomyia, ficalbia, mimomyia, hodgesia, coquillettidia, mansonia, and uranotaenia.* Southeast Asian Journal of Tropical Medicine and Public Health, 2006. **37**(S1): p. 1.
69. Forattini, O.P., *Culicidologia médica: identificação, biologia, epidemiologia Vol. 2.* 1996: Edusp.
70. Soares Gil, L.H., et al., *Evaluation of Mansonia spp. infestation on aquatic plants in lentic and lotic environments of the Madeira River basin in Porto Velho, Rondônia, Brazil.* Journal of the American Mosquito Control Association, 2021. **37**(3): p. 143-151.
71. Smithburn, K., A. Haddow, and J. Gillett, *Rift Valley fever. Isolation of the virus from wild mosquitoes.* British journal of experimental pathology, 1948. **29**(2): p. 107.
72. McIntosh, B., *Rift Valley fever: 1. Vector studies in the field.* Journal of the South African Veterinary Association, 1972. **43**(4): p. 391-395.
73. Hopkins, G.H., *Mosquitoes of the Ethiopian region. I. Larval bionomics of mosquitoes and taxonomy of Culicine larvae.* 1952.
74. Hartberg, W. and E. Gerberg, *Laboratory colonization of Aedes simpsoni (Theobald) and Eretmapodites quinquevittatus Theobald.* Bulletin of the World Health Organization, 1971. **45**(6): p. 850.
75. Cêtre-Sossah, C., et al., *Evidence of Eretmapodites subsimplicipes and Aedes albopictus as competent vectors for Rift Valley fever virus transmission in Mayotte.* Acta Tropica, 2023. **239**: p. 106835.
76. Christensen, B.M., et al., *Laboratory studies of transovarial transmission of trivittatus virus by Aedes trivittatus.* The American Journal of Tropical Medicine and Hygiene, 1978. **27**(1 Pt 1): p. 184-186.
77. Taylor, D., et al., *California group arboviruses in Florida. Host-vector relations.* 1971.
78. Tesh, R.B., *Experimental studies on the transovarial transmission of Kunjin and San Angelo viruses in mosquitoes.* 1980.
79. Tesh, R.B. and D.A. Shroyer, *The mechanism of arbovirus transovarial transmission in mosquitoes: San Angelo virus in Aedes albopictùs.* The American journal of tropical medicine and hygiene, 1980. **29**(6): p. 1394-1404.
80. Tesh, R.B. and M. Cornet, *The location of San Angelo virus in developing ovaries of transovarially infected Aedes albopictus mosquitoes as revealed by fluorescent antibody technique.* The American Journal of Tropical Medicine and Hygiene, 1981. **30**(1): p. 212-218.
81. Mitchell, C.J., *Vector competence of North and South American strains of Aedes albopictus for certain arboviruses: a review.* J Am Mosq Control Assoc, 1991. **7**(3): p. 446-451.
82. Mitchell, C., et al., *Arboviruses associated with mosquitoes from nine Florida counties during 1993.* Journal of the American Mosquito Control Association-Mosquito News, 1996. **12**(2): p. 255-262.
83. Le Duc, J.W., et al., *Ecology of California encephalitis viruses on the Del Mar Va Peninsula. II. Demonstration of trans-ovarial transmission.* 1975.
84. Chamberlain, R., et al., *Vector studies in the St. Louis encephalitis epidemic, Tampa Bay area, Florida, 1962.* The American Journal of Tropical Medicine and Hygiene, 1964. **13**(3): p. 456-461.
85. Elbadry, M.A., et al., *Diversity and Genetic Reassortment of Keystone Virus in Mosquito Populations in Florida.* The American Journal of Tropical Medicine and Hygiene, 2023. **108**(6): p. 1256.
86. Mutisya, J., et al., *Evaluating the vector competence of Aedes simpsoni sl from Kenyan coast for Ngari and Bunyamwera viruses.* Plos one, 2021. **16**(7): p. e0253955.
87. Zeller, H., et al., *Ngari virus (Bunyaviridae: Bunyavirus). First isolation from humans in Senegal, new mosquito vectors, its epidemiology.* Bulletin de la Societe de Pathologie Exotique (1990), 1996. **89**(1): p. 12-16.
88. Gordon, S.W., et al., *Arbovirus isolations from mosquitoes collected during 1988 in the Senegal River basin.* American Journal of Tropical Medicine and Hygiene, 1992. **47**: p. 742-742.
89. Tauro, L.B., et al., *First isolation of Bunyamwera virus (Bunyaviridae family) from horses with neurological disease and an abortion in Argentina.* The Veterinary Journal, 2015. **206**(1): p. 111-114.

90. Odhiambo, C., et al., *Vector competence of selected mosquito species in Kenya for Ngari and Bunyamwera viruses.* Journal of medical entomology, 2014. **51**(6): p. 1248-1253.
91. Lozach, P.-Y., et al., *Entry of bunyaviruses into mammalian cells.* Cell host & microbe, 2010. **7**(6): p. 488-499.
92. Hubálek, Z., *Mosquito-borne viruses in Europe.* Parasitology research, 2008. **103**: p. 29-43.
93. Pinheiro, F.P., A.P. Travassos da Rosa, and J.F. Travassos da Rosa, *Oropouche virus. I. A review of clinical, epidemiological, and ecological findings.* 1981.
94. Dias, H.G., F.B. Dos Santos, and A. Pauvolid-Corrêa, *An overview of neglected orthobunyaviruses in Brazil.* Viruses, 2022. **14**(5): p. 987.
95. Saeed, M.F., et al., *Jatobal virus is a reassortant containing the small RNA of Oropouche virus.* Virus research, 2001. **77**(1): p. 25-30.
96. Aguilar, P.V., et al., *Iquitos virus: a novel reassortant Orthobunyavirus associated with human illness in Peru.* PLoS neglected tropical diseases, 2011. **5**(9): p. e1315.
97. Fulhorst, C.F., et al., *Geographic distribution and serologic and genomic characterization of Morro Bay virus, a newly recognized bunyavirus.* The American journal of tropical medicine and hygiene, 1996. **54**(6): p. 563-569.
